# Nomogram to predict risk of resistance to intravenous immunoglobulin in children hospitalized with Kawasaki disease in Eastern China

**DOI:** 10.1080/07853890.2022.2031273

**Published:** 2022-01-31

**Authors:** Hongbiao Huang, Jiaqi Jiang, Xiaosong Shi, Jie Qin, Jinfeng Dong, Lei Xu, Chengcheng Huang, Ying Liu, Yiming Zheng, Miao Hou, Qin Shen, Bihe Zeng, Guanghui Qian, Fang Yang, Haitao Lv

**Affiliations:** a Department of Pediatrics, Institute of Pediatric Research, Children's Hospital of Soochow University, P.R. China; b Department of Pediatrics, Fujian Provincial Hospital, Fujian Provincial Clinical College of Fujian Medical University, Fuzhou, P.R. China; c Department of Hematology and Rheumatology, the First Affiliated Hospital of Fujian Medical University, Fuzhou, P.R. China

**Keywords:** Kawasaki disease, LASSO regression, nomogram, prediction model, intravenous immunoglobulin resistance

## Abstract

**Objective:**

We aimed to develop a nomogram to predict risk of resistance to intravenous immunoglobulin (IVIG) in children with Kawasaki disease in eastern China.

**Methods:**

We retrospectively analysed the data of children with Kawasaki disease who received IVIG during hospitalisation at Soochow University Affiliated Children’s Hospital. IVIG resistance was defined as recrudescent or persistent fever ≥36 h after the end of the IVIG infusion. Baseline variables were analysed using least absolute shrinkage and selection operator (LASSO) to identify the predictors of IVIG resistance, which were then used to construct a predictive nomogram. Calibration curve and area under the receiver operating characteristic curve (AUC) were used to evaluate the performance of the model. The predictive nomogram was validated on test sets of external data and prospective data.

**Results:**

Between January 2015 and December 2020, 1293 Kawasaki disease patients were hospitalized in Soochow University Affiliated Children’s Hospital. Among them, 72 (5.57%) showed IVIG resistance. LASSO identified haemoglobin, percentage of neutrophils, C-reactive protein level, platelet count, serum albumin, serum sodium, serum alkaline phosphatase, coronary artery damage, and complete Kawasaki disease as risk factors for IVIG resistance. The nomogram constructed using these factors showed satisfactory discriminatory power (AUC, 0.75), and sensitivity (0.74) and specificity (0.64). In the external data and prospective data, the AUC was 0.66 and 0.83, respectively, the sensitivity was 0.86 and 1, respectively, and the specificity was 0.49 and 0.60, respectively.

**Conclusions:**

The predictive nomogram constructed using nine factors associated with IVIG resistance in children with Kawasaki disease could be a useful tool for identifying patients likely to show IVIG resistance. This nomogram may help reduce the risk of coronary artery lesions.

**Key Messages**
1. None of the IVIG resistance scoring systems has shown consistently good performance in previous studies. Tools to predict the risk of IVIG resistance in eastern China are lacking.
2. In our series, haemoglobin level, percentage of neutrophils, platelet count, coronary artery damage, incomplete Kawasaki disease, and CRP, serum albumin, serum sodium, and serum alkaline phosphatase levels were risk factors of IVIG resistance in hospitalized children in the eastern China cities of Suzhou and Fuzhou.
3. We propose an easy-to-use nomogram to predict the risk factors of IVIG resistance in hospitalized children.

## Introduction

Kawasaki disease is an acute-onset self‐limiting vasculitis that affects infants and children. Without timely treatment, up to 25% of patients develop coronary artery lesions [1]. Intravenous immunoglobulin (IVIG) is the most effective initial treatment for acute symptoms, but 10–20% of patients will have IVIG resistance and remain at risk for developing coronary artery lesions [2]. The mechanism of action of IVIG in Kawasaki disease is still not understood [3], and the factors influencing IVIG resistance have not yet been identified. In patients who show IVIG resistance, subsequent treatment is not as effective [4,5]. The addition of corticosteroid to initial IVIG therapy can significantly decrease coronary artery lesions in Kawasaki disease patients with s predicted high risk of nonresponse to IVIG alone [6]. Therefore, early prediction of the risk of IVIG resistance is important.

There are several Japanese scoring systems for predicting IVIG resistance, including the Kobayashi, Egami, and Sano scoring systems [7–9]. However, these scoring systems do not perform well in non-Japanese populations [10,11], which may be attributable to racial and other differences between the study populations [12]. Thus, it is essential to develop reliable models for different populations. The aim of this study was to (1) identify the predictors of IVIG resistance in children hospitalised with Kawasaki disease in the eastern China cities of Suzhou and Fuzhou and (2) to develop and validate a predictive nomogram for use in the clinic.

## Patients and methods

### Patient selection and data collection

We searched the electronic medical records of the Affiliated Children’s Hospital of Soochow University and the Fujian Provincial Hospital to identify all patients admitted with first diagnosis of Kawasaki disease between January 2015 and December 2020. Patients were eligible for inclusion if they had been diagnosed with incomplete or complete Kawasaki disease by the American Heart Association (AHA) criteria [13,14]. According to the AHA criteria, complete Kawasaki disease is diagnosed when patients have fever for ≥5 days plus at least four of the following five clinical features: rash, bilateral conjunctive injection, cervical lymphadenopathy, changes in the extremities, and oral mucosal changes. Meanwhile, incomplete Kawasaki disease is diagnosed when ≥5 days of fever is accompanied by two or three of the above clinical features plus C-reactive protein (CRP) ≥30 mmol/L or erythrocyte sedimentation rate (ESR) ≥40 mm/h. We excluded patients who had received IVIG at another hospital (*n* = 16), those with history of previous hospitalization for Kawasaki disease (*n* = 57), and patients who had not received IVIG as initial treatment (*n* = 20). Any cases involving the use of steroids or immunosuppressive drugs before or in combination with IVIG treatment were also excluded.

Data on baseline characteristics, laboratory test results, echocardiography, and clinical findings during hospitalization were collected from the hospital records. Diagnosis of coronary artery lesions was based on echocardiography changes or the presence of a set of suspicious laboratory criteria (as specified in the AHA guidelines).

Most patients received IVIG 2 g/kg as a single dose according to the AHA guidelines [14]. In patients who experienced mild IVIG-related adverse events, including headache, flushing, malaise, arthralgia, IVIG treatment at 1 g/kg per day for two consecutive days was attempted. Aspirin 30–50 mg/kg was given as recommended in the AHA guidelines [14]. A patient was considered afebrile when body temperature remained below 37.5 °C for more than 24 h [7]. The definition of body temperature for determining resistance is fever that cannot resolve within 36 h after completion of IVIG infusion [14].

Data from the Children’s Hospital of Soochow University constituted the training set (*n* = 1293, IVIG resistance = 5.57%), while data collected from Fujian Provincial Hospital (*n* = 205, IVIG resistance = 7.33%) constituted the external validation test set. Prospective validation was performed on data collected in Soochow University Affiliated Children’s Hospital from January 2021 to April 2021 (*n* = 209, IVIG resistance = 2.96%).

This study was performed in accordance with the Declaration of Helsinki and was approved by the Ethics Committee of Children’s Hospital of Soochow University, Suzhou (Suzhou, China; approval no. 2020CS075). The study protocol was explained to the participants and their parents and written informed consent was obtained.

### Evaluated variables

Variables considered for constructing the model included demographic characteristics (sex, age); clinical features (incomplete Kawasaki disease, IVIG resistance); and laboratory tests results (CRP, haemoglobin, white blood cell count, percentage of neutrophils, platelet count, serum albumin, serum aspartate aminotransferase [AST], serum alanine aminotransferase [ALT], serum total bilirubin, serum lactate dehydrogenase [LDH], serum alkaline phosphatase, serum sodium, serum calcium, urine white blood cell count).

Laboratory tests were performed on the first day of hospitalization. The same reagents were used for all patients. Cardiac colour Doppler ultrasound examination was performed by paediatric echocardiographers using IE33 and IE Elite machines (Philips, Amsterdam, the Netherlands). The ultrasound reports were reviewed by experienced paediatric echocardiographers, each with >5 years’ experience in paediatric echocardiography. Coronary artery diameter was classified according to the Japanese Ministry of Health criteria as abnormal when the internal lumen diameter was ≥3 mm in children below five years or ≥4 mm in children above five years [15].

### Statistical methods

Data were first entered into SPSS 24.0 (IBM Corp., Armonk, NY, USA), with multiple imputation used to handle missing data. It is known that, for any variable, a large number of missing values could lead to bias, but the acceptable proportion of missing values is not defined. In this study, we excluded two variables with more than 50% missing values; these were serum calcium and urine white blood cell count. The remaining 16 variables (Table 1) were subjected to further analysis. Specific data related to missing values and imputation methods are presented in Supplement 1. Subsequent statistical analysis was performed using R 4.0.4 (https://www.R-project.org). The least absolute shrinkage and selection operator (LASSO) method was used to select the predictors of IVIG resistance [16]. Variables with non-zero coefficients were selected in the LASSO regression model [17]. As one of the classic algorithms of machine learning, Random Forest shows high accuracy in disease risk prediction and diagnosis. We also performed a Random Forest analysis and compared the results to those obtained with Lasso regression [18]. The regression coefficients of the nine independent predictors thus identified in the training set were used for construction of the predictive model. Calibration curves were plotted to assess the calibration of the nomogram. We used some metrics—AUC, 95% confidence interval (CI), sensitivity, and specificity—to evaluate the performance of the model in the training set. The data from Fujian Provincial Hospital were used for external verification, and the data from January 2021 to April 2021 from the Children’s Hospital of Soochow University were used for prospective validation.

**Table 1.** Comparison of clinical characteristic between the IVIG-responsive and IVIG-resistant groups (*n* = 1707).

Variable	Training set		Test set			
	Kawasaki disease patients admitted in Soochow University Affiliated Children’s Hospital from January 2015 to December 2020 (*n* = 1293)		Kawasaki disease patients admitted in Fujian Provincial Hospital from January 2015 to December 2020 (*n* = 205)		Kawasaki disease patients admitted in Soochow University Affiliated Children's Hospital from January 2021 to April 2021 (*n* = 209)	
	IVIG-responsive (*n* = 1221)	IVIG-resistant (*n* = 72)	IVIG-responsive (*n* = 191)	IVIG-resistant (*n* = 14)	IVIG-responsive (*n* = 203)	IVIG-resistant (*n* = 6)
Male:Female	738:483 (1.53:1)	42:30 (1.4:1)	119:72 (1.65:1)	13:1 (13:1)	124:79 (1.57:1)	5:1 (5:1)
Age in months, mean (range)	26.34 (1-145)	28.3 (2-89)	30.07 (1-144)	46.57 (5-144)	37.94 (2-158)	39.83 (3-65)
WBC count, mean ± SD (median), ×10^9^/L	14.96 ± 5.31 (14.33)	15.62 ± 7.47 (14.25)	13.34 ± 5.74 (12.5)	13.5 ± 6.38 (13.3)	15.25 ± 7.66 (14.04)	13.77 ± 2.22 (14.08）
% neutrophils, mean ± SD (median)	63.84 ± 15.76 (64.7)	74.3 ± 14.15 (76.5)	58.57 ± 18.31 (60.3)	64.43 ± 19.85 (62.7)	65.09 ± 18.56 (67.64)	78.09 ± 13.63 (80.78)
Haemoglobin, mean ± SD (median), g/L	109.13 ± 10.98 (109)	105.6 ± 13.72 (105)	112.81 ± 13.27 (112)	106.43 ± 16.09 (101)	109.76 ± 11.28 (109)	101.83 ± 13.69 (107)
Platelet count, mean ± SD (median), ×10^9^/L	370.98 ± 127.15 (357)	368.29 ± 154.94 (346)	378.54 ± 152.32 (362)	350.21 ± 114.76 (336)	340.17 ± 103.09 (327)	366.67 ± 53.93 (362)
CRP, mean ± SD (median), mg/L	76.49 ± 54.35 (65.36)	105.76 ± 58.82 (96.59)	58.62 ± 46.86 (51.4)	80.74 ± 57.53 (62)	68.04 ± 47.07 (59.22)	96.53 ± 32.92 (102.57)
Serum albumin, mean ± SD (median), g/L	38.67 ± 3.69 (39)	36.91 ± 4.77 (37.15)	36.3 ± 5.02 (36)	32.21 ± 5.18 (32.5)	39.44 ± 4.04 (39.5)	35.62 ± 8.01 (38.8)
Serum sodium, mean ± SD (median), mmol/L	134.66 ± 3.14 (135)	134.03 ± 3.45 (134)	135.53 ± 3.51 (135)	134.71 ± 3.6 (134)	135.26 ± 2.76 (136)	136.67 ± 1.03 (137)
ALT, mean ± SD (median), U/L	56.47 ± 120.22 (22.9)	68.01 ± 90 (26.2)	71.29 ± 105.94 (28)	45.21 ± 37.91 (37)	52.56 ± 81.49 (20.3)	102.6 ± 144.52 (28.5)
AST, mean ± SD (median), U/L	51.48 ± 99.74 (32)	57.73 ± 65.82 (35.7)	53.31 ± 93.01 (32)	42.29 ± 20.91 (34)	54.99 ± 84.91 (33.8)	156.1 ± 186.4 (58.9)
LDH, mean ± SD (median), U/L	420.55 ± 145.16 (402.7)	418.17 ± 115.76 (411.2)	381.64 ± 310.03 (291)	441.36 ± 240.15 (321)	405.01 ± 140.98 (382.5)	395.52 ± 168.50 (360.15)
TBil, mean ± SD (median), μmol/L	12.29 ± 34.15 (5.5)	16 ± 19.35 (7.5)	9.86 ± 12.57 (6.08)	11.69 ± 11.87 (9.61)	8.14 ± 9.94 (5.6)	12.3 ± 16.39 (6.05)
ALP, mean ± SD (median), U/L	189.65 ± 84.28 (178)	211.88 ± 104.21 (191.5)	203.62 ± 139.44 (173)	174.93 ± 60.2 (164.5)	174.24 ± 51.85 (160)	211.83 ± 76.98 (197.5)
Coronary artery lesions:Coronary artery no lesions	944:277 (3.41:1)	60:12 (5:1)	109:82 (1.33:1)	7:7 (1:1)	176:27 (6.52:1)	5:1 (5:1)
Complete Kawasaki disease:Incomplete Kawasaki disease	963:258 (3.73:1)	66:6 (11:1)	162:29 (5.59:1)	12:2 (6:1)	196:7 (28:1)	6:0

IVIG: intravenous immunoglobulin; WBC: white blood cell; SD: standard deviation; CRP: C-reactive protein; ATL: serum alanine aminotransferase; AST: serum aspartate aminotransferase; LDH: serum lactate dehydrogenase; TBil: serum total bilirubin; ALP: serum alkaline phosphatase.

## Results

### Patients’ characteristics

A total of 1707 children with Kawasaki disease were included in this study. As the training set for construction of the LASSO regression model, we used the data of 1293 children who were treated from January 2015 to December 2020 at the Children’s Hospital of Soochow University. The test set included two parts: The data of 205 children with Kawasaki disease treated between January 2015 and December 2020 at the Fujian Provincial Hospital were used for external verification of the model, while the data of 209 children treated between January 2021 and April 2021 at the Children’s Hospital of Soochow University were used for prospective validation.

Of the 1293 patients, 92 (5.39%) had IVIG resistance. IVIG resistance was the dependent variable, and the 16 predictive variables (Table 1) were the independent variables. In addition, a total of 208 healthy children were included in the study to compare some predictive variables of KD children with age-matched healthy children.

### Variable selection and development of the predictive nomogram

**Figure 1.**
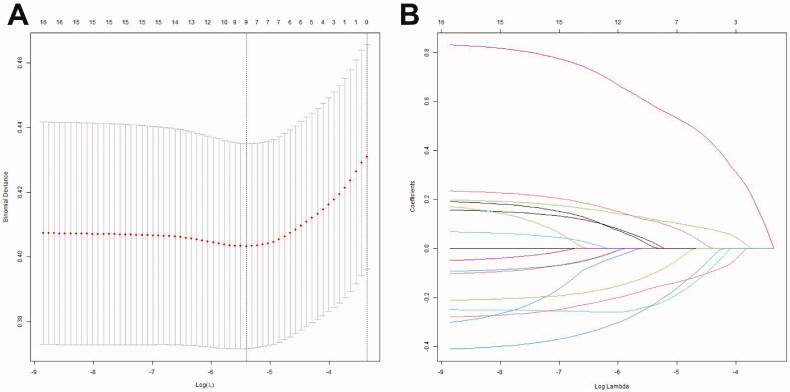
Selection of predictors using the LASSO logistic regression model (coronary artery lesions were evaluated using the internal lumen diameter). (A) Optimal parameter (lambda) selection in the LASSO model used fivefold cross-validation via minimum criteria. The partial likelihood deviance (binomial deviance) curve was plotted versus log(lambda). Dotted vertical lines were drawn at the optimal values by using the minimum criteria and the 1 SE of the minimum criteria (the 1-SE criteria). (B) LASSO coefficient profiles of the 16 features. A coefficient profile plot was produced against the log(lambda) sequence. The optimal lambda resulted in nine features with non-zero coefficients. LASSO: least absolute shrinkage and selection operator; SE: standard error.

**Figure 2.**
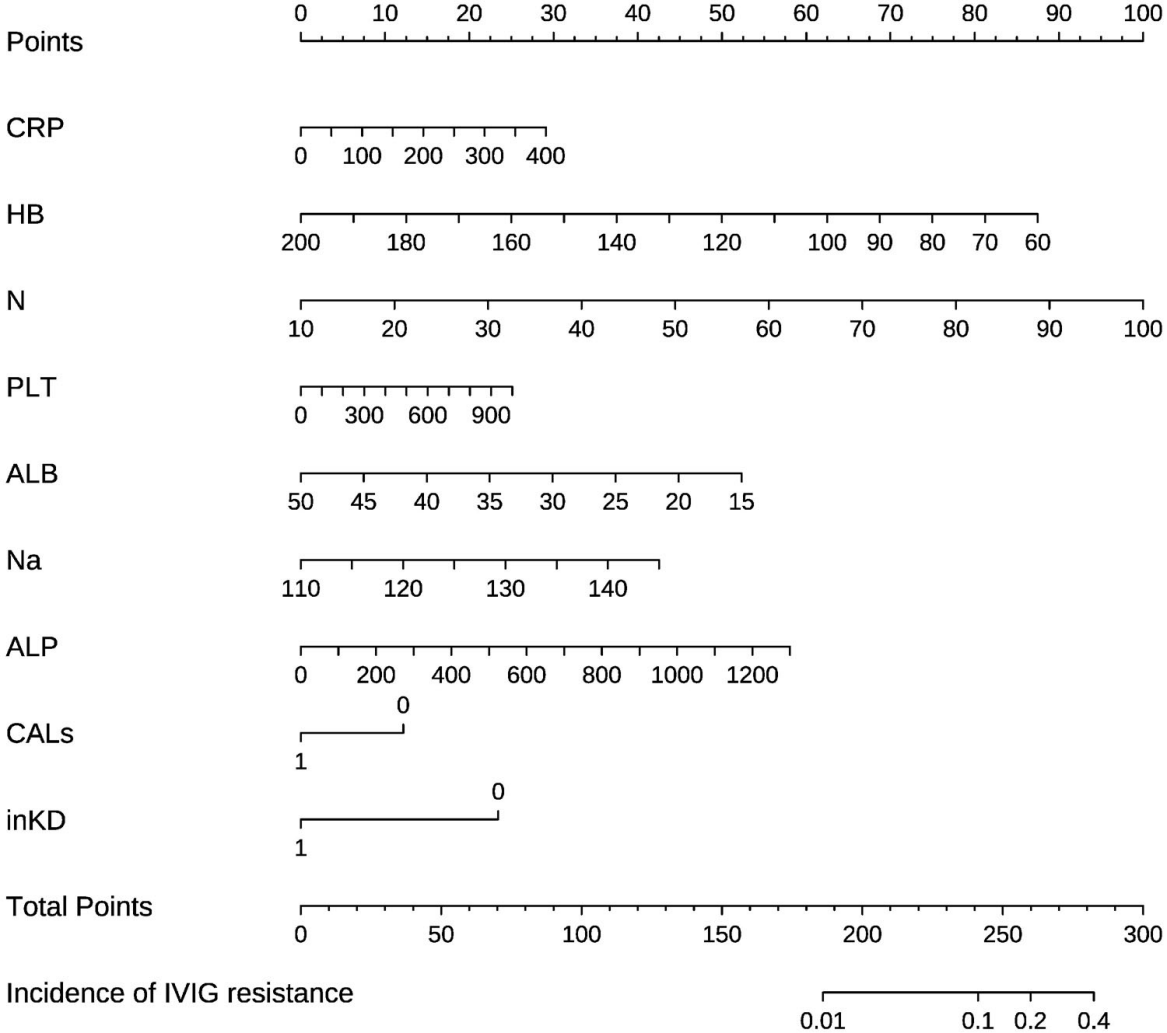
Nomogram developed for predicting IVIG resistance. The nomogram incorporated haemoglobin, percentage of neutrophils, C-reactive protein level, platelet count, serum sodium, serum albumin, serum alkaline phosphatase, coronary artery damage, and complete Kawasaki disease. IVIG: intravenous immunoglobulin; CRP: C-reactive protein; HB: haemoglobin; N: percentage of neutrophils; Na: serum sodium; ALB: albumin; ALP: alkaline phosphatase; CALs: coronary artery lesions; inKD: incomplete Kawasaki disease.

From among the 16 predictive variables identified in the LASSO regression model (Figure 1(A,B)), we selected nine variables with optimal lambda (λ = 0.004483563);. these variables were haemoglobin, percentage of neutrophils, platelet count, CRP, serum albumin, serum sodium, serum alkaline phosphatase, coronary artery damage, and incomplete Kawasaki disease. Table 2 lists the regression coefficients of each variable, as well as the relative risk (with 95% confidence interval) calculated in logistic regression analysis. The nine independent predictors were used to construct the nomogram (Figure 2).

**Table 2.** Predictive factors for IVIG resistance in patients with Kawasaki disease.

Variables	Coefficients	*p* Value
Intercept	−8.7414	.1574
Haemoglobin	−0.0271	.0251
%Neutrophils	0.0483	<.0001
Platelet count	0.0011	.2759
C-reactive protein	0.0032	.1714
Serum albumin	−0.0649	.0711
Serum sodium	0.0528	.2321
Alkaline phosphatase	0.0019	.0946
Coronary artery lesions	−0.5282	.1138
Incomplete Kawasaki	−1.0183	.0217

### Performance of nomogram in the training set

**Figure 3.**
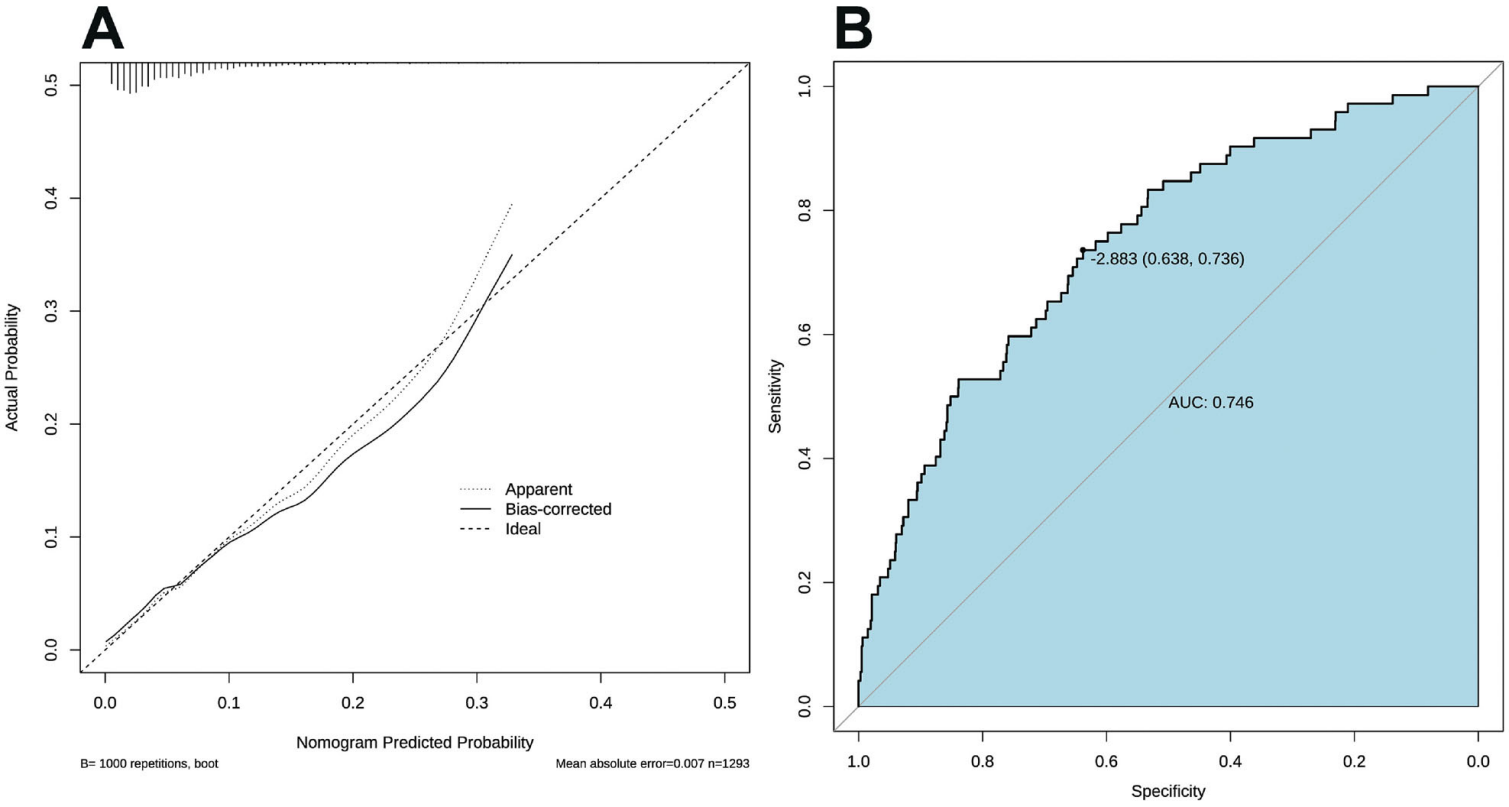
Calibration curves and receiver operator characteristic curves of the nomogram prediction in the training set. (A) The x-axis represents the predicted IVIG-resistance risk. The y-axis represents the actual diagnosed IVIG resistance. The diagonal dotted line represents a perfect prediction by an ideal model. The solid line represents the performance of the nomogram, with closer fit to the diagonal dotted line representing a better prediction. (B) Receiver operator characteristic curves for IVIG-resistance prediction model.

The calibration curve of the nomogram for the prediction of IVIG resistance risk demonstrated good agreement in the training set (Figure 3(A)). The AUC was 0.75 (95% CI: 0.69–0.80), and the sensitivity and specificity were 0.74 and 0.64, respectively (Figure 3(B)).

**Figure 4.**
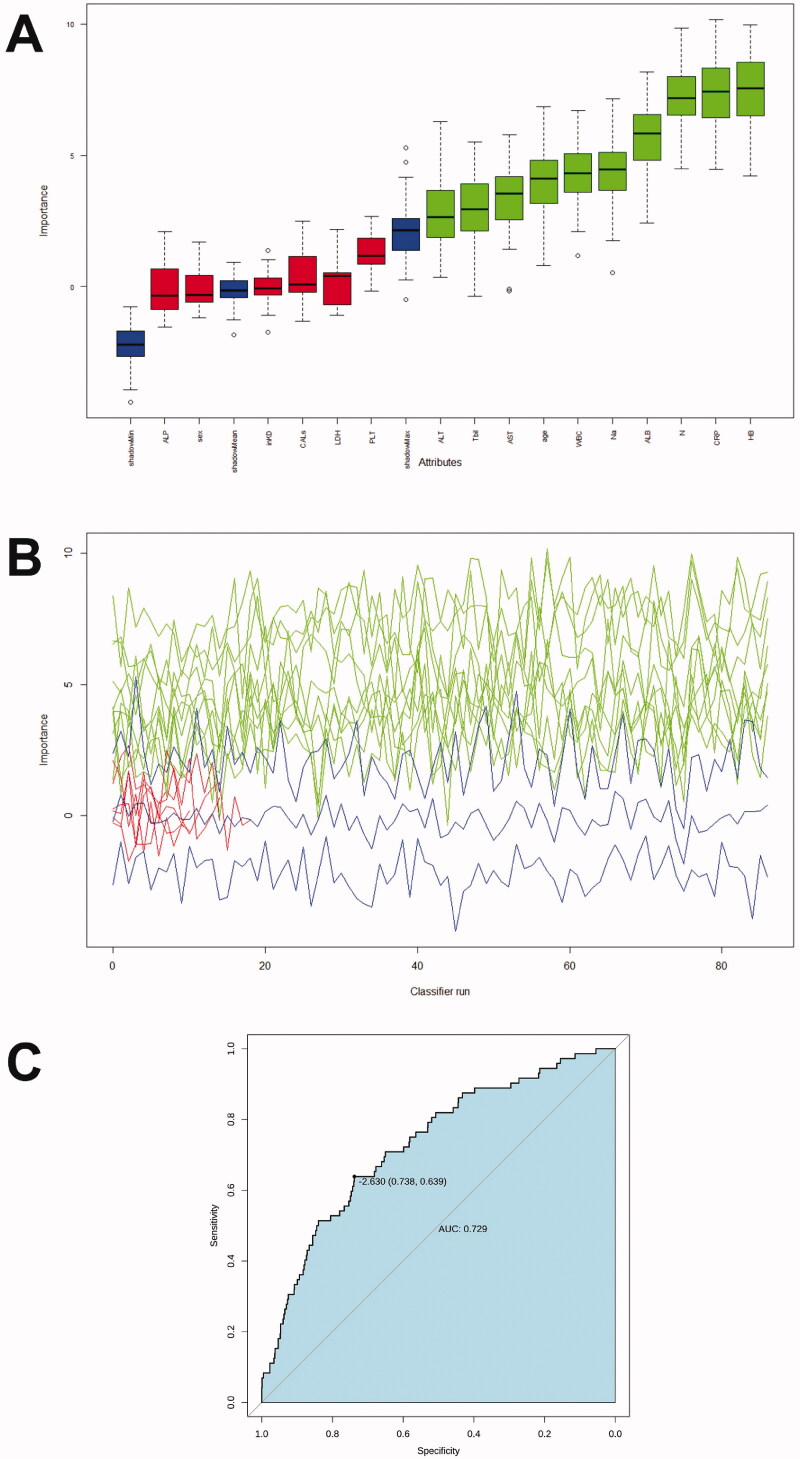
Feature selection and ranking by Random Forest. (A) The plot shows a boxplot of all the attributes plus the minimum, average, and maximum shadow scores. Variables with boxplots in green are important variables confirmed by Random Forest. Boxplots in red indicate that the variables were rejected. (B) History of decisions regarding the rejection or acceptance of features by Random Forest in 500 Boruta function runs. (C) Receiver operator characteristic curves for the IVIG-resistance prediction model calculated by Random Forest.

Random Forest was then performed. The number of decision trees constructed in this study was 500, and four variables were randomly selected for each decision tree node. The Random Forest selected or excluded variables according to the feature importance. The model was then verified in the training dataset (from 70% of training data) and testing dataset (from 30% of training data) with the following parameters serving as assessment tools: area under the curve (AUC), sensitivity, specificity. Ten variables were included in the study, including age, white blood cell count, haemoglobin level, percentage of neutrophils, and serum albumin, AST, ALT, serum total bilirubin, serum sodium, and CRP level (Figure 4(A,B)). These variables were used to create a new model. The AUC of the new model was 0.73 (95% CI: 0.67–0.79), and the sensitivity and specificity were 0.64 and 0.74, respectively (Figure 4(C)). The selection process of these variables is presented in Supplement 2.

In comparisons between the two models, the AUC area of the model calculated by LASSO regression was better, but the difference was not statistically significant (*p* = .24). Since the LASSO regression model included fewer variables and was more conducive to clinical application, we chose the LASSO regression model for the rest of the study.

### Validation of the nomogram in the test set

**Figure 5.**
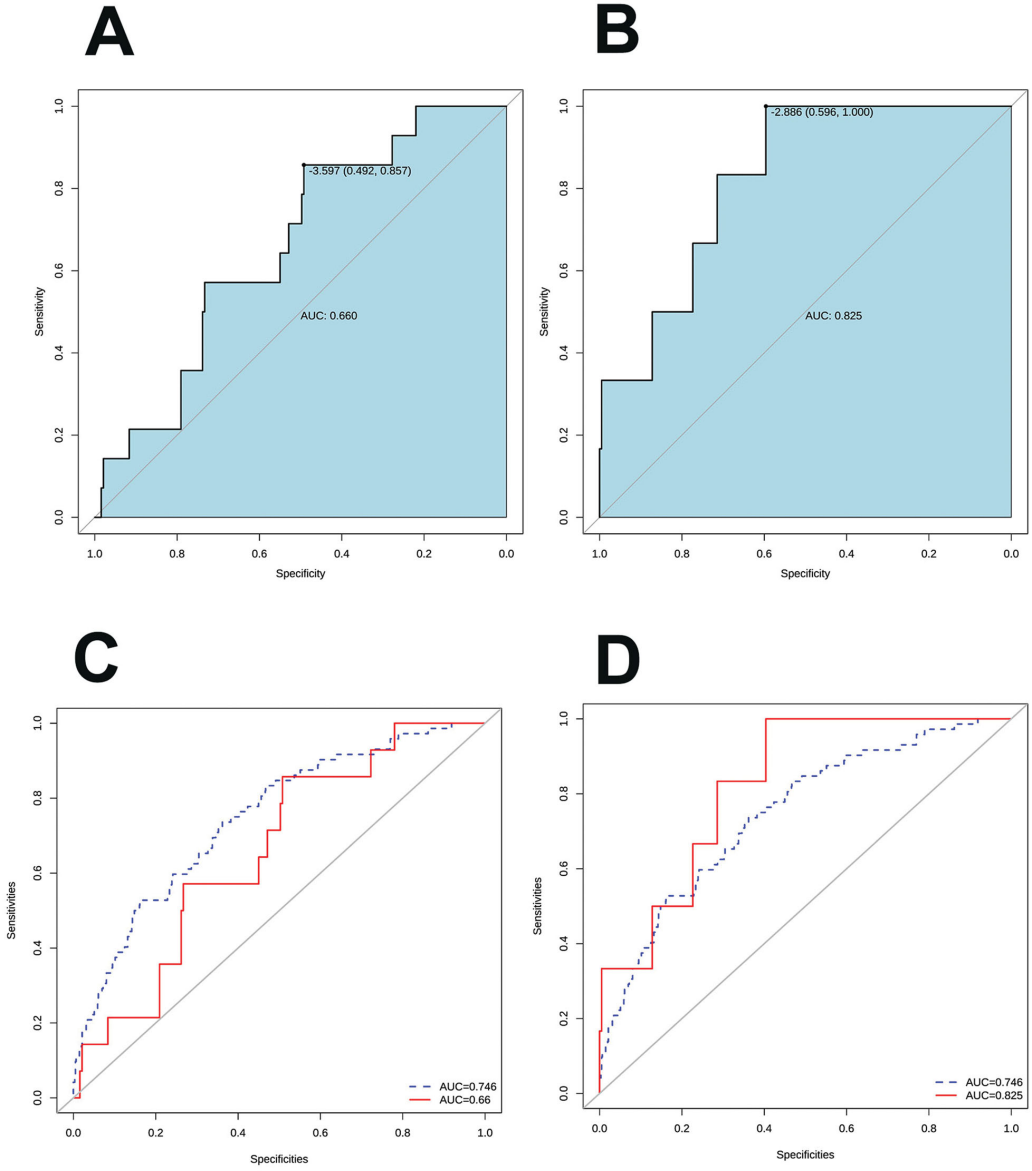
Receiver operator characteristic curves of the nomogram prediction in the test set. (A) Receiver operator characteristic curve was used to validate the performance of the nomogram in external data. (B) Receiver operator characteristic curve was used to validate the performance of the nomogram in prospective data. (C) Comparison of the area under the receiver operator characteristic curves of the external data (solid line) and training set (dotted line). (D) Comparison of the area under the receiver operator characteristic curves of the prospective data (solid line) and training set (dotted line).

We used both external data and prospective data to verify the model. In the external data (from Fujian Provincial Hospital), the AUC was 0.66 (95% CI: 0.53–0.79), and the sensitivity and specificity were 0.86 and 0.49, respectively (Figure 5(A)). In the prospective data (from the Children’s Hospital of Soochow University), the AUC was 0.83 (95% CI: 0.69–0.96), and the sensitivity and specificity were 1 and 0.60, respectively (Figure 5(B)). Figure 5(C,D) show the comparison between external validation and prospective data validation, and the AUC of the training set.

### Comparison of the levels of serum ALP in children of different ages

In our study, serum alkaline phosphatase (ALP) was a special risk factor of IVIG resistance. Due to bone growth and development, the levels of serum ALP were related to the age of children. To elucidate the differences in serum ALP levels between IVIG-responsive and age-matched IVIG-resistant patients, we classified patient ages as follows according to the Nelson Textbook of Paediatrics [19]: infancy (0–1 years), toddlerhood (1 ∼ 3 years), pre-school (3 ∼ 6 years), school age (6 ∼ 12 years), and adolescence (12 ∼ 20 years). The levels of serum ALP did not reach significance in our study (Table 3). Furthermore, we also compared serum ALP levels between KD patients and age-matched healthy children. A total of 208 healthy children were included in our study. The results showed that KD patients had higher serum ALP levels than age-matched healthy children during infancy, toddlerhood, and pre-school age (Table 4); the corresponding intergroup differences were significant.

**Table 3.** Comparison of serum alkaline phosphatase levels between the IVIG-responsive and IVIG-resistant groups in different age stage.

Variable	ALP, mean ± SD (median), U/L		
Age classic stage	IVIG-responsive	IVIG-resistant	*p* Value
Infancy (0 ∼ 12 months) (*N*)	178.1 ± 4.5 (361)	181.6 ± 14.1 (21)	.76
Toddlerhood (12.1 ∼ 36 months) (*N*)	192.8 ± 3.4 (577)	205.2 ± 12.8 (30)	.3
Pre-school (36.1 ∼ 72 months) (*N*)	200.7 ± 5.5 (225)	258.9 ± 42.1 (17)	.07
School age (72.1 ∼ 144 months) (*N*)	186.2 ± 14.8 (57)	211.0 ± 15.9 (4)	.08
Adolescence (144.1 ∼ 240 months) (*N*)	199 (1)	0	–

IVIG: intravenous immunoglobulin; ALP: serum alkaline phosphatase.

**Table 4.** Comparison of serum alkaline phosphatase levels between the KD patients and age-matched healthy children.

Variable	ALP, mean ± SD (median), U/L		
Age classic stage	Health children	KD patients	*p* Value
Infancy (0 ∼ 12 months) (*N*)	146.1 ± 4.8 (60)	178.3 ± 4.3 (382)	<.001
Toddlerhood (12.1 ∼ 36 months) (*N*)	161.0 ± 4.9 (94)	193.4 ± 3.2 (607)	<.001
Pre-school (36.1 ∼ 72 months) (*N*)	166.6 ± 6.7 (41)	204.8 ± 5.9 (242)	.007
School age (72.1 ∼ 144 months) (*N*)	157.8 ± 25.2 (13)	188.5 ± 13.9 (61)	.07
Adolescence (144.1 ∼ 240 months) (*N*)	0	199 (1)	–

ALP: serum alkaline phosphatase; KD: Kawasaki Disease.

### Comparison of Z-score-correlated ROC curves and internal lumen diameter-correlated ROC curves

Z scores are being used more widely for classifying coronary artery involvement [14]. We also compared the difference between Z-score correlated ROC curves and internal lumen diameter-correlated ROC curves. CALs were recalculated by echocardiography and were defined by Z scores according to AHA guidelines as follows [14]: 1. No involvement: <2; 2. Dilation only: 2 to <2.5, or if initially <2, a decrease in Z score during follow-up ≥ 1; 3. Small aneurysm: ≥2.5 to <5; 4. Medium aneurysm: ≥5 to <10, and absolute dimension <8 mm; 5. Large or giant aneurysm: ≥10. LASSO regression reidentified haemoglobin level, percentage of neutrophils, C-reactive protein, serum albumin, and serum alkaline phosphatase levels, and complete Kawasaki disease as risk factors for IVIG resistance. The selection process of these variables is presented in Supplement 3. The new nomogram constructed using these factors showed similar discriminatory power (AUC, 0.74), sensitivity (0.74), and specificity (0.66) (Supplement 4). Then, DeLong's test was used to compare Z-score-correlated ROC curves and internal lumen diameter-correlated ROC curves, and the result showed no statistical difference (*p*-value = .66).

## Discussion

In our research, by retrospective analysis of the data of 1293 patients from eastern China, we identified nine variables as significant independent predictors of IVIG resistance. The predictive nomogram based on these nine factors showed good performance in the training set, with AUC, sensitivity, and specificity of 0.75, 0.74, and 0.64, respectively.

Notably, only 92 (5.4%) patients did not respond to the initial IVIG treatment, which was lower than the proportions reported in previous (15%∼25%) [20]. According to many previous studies, the incidence of IVIG resistance seems to be lower in China than in other countries. Yang et al. [21] reported that the incidence of IVIG resistance was 6% in Beijing, the capital of China. Tang et al. [12], Xie et al. [22], and Wei et al. [23] reported that the incidence of IVIG resistance was 5.1%, 10%, and 6.8%, respectively, in Suzhou, Hangzhou, and Shanghai, which are three major cities in eastern China. This might be partly explained by our use of IVIG within the 5–9 days of illness, since early administration of IVIG before the fourth day was widely accepted a risk factor for IVIG resistance according to Kobayashi score [7].

Another noteworthy result in our study is that the incidence of CALs was similar in IVIG-responsive (22.9%) and IVIG-resistance (21.7%) patients, which was different from the results of previous reports [2]. The increased incidence of KD in certain population groups suggests the involvement of genetic factors in susceptibility to KD [24]. Yuan et al. [25] reported that the rate of CALs in IVIG-effective cases (47.4%) was even higher than that in IVIG-resistance cases (28.9%). Chantasiriwan et al. [26] reported that the incidence of initial CALs in IVIG-effective cases was 41%. This rate was similar to that in the IVIG-resistance cases (42%). However, after 6 ∼ 8 weeks, the incidence of CALs in IVIG-sensitive cases (24%) was significantly lower than that in IVIG-resistance cases (35%). In our study, echocardiography was performed at the acute stage, which may have led to an increase in CALs in the IVIG-effective cases. Therefore, a higher incidence of CALs in IVIG-effective cases may be due to differences in genetic background and early detection time. We also compared Z-score-correlated ROC curves and internal lumen diameter-correlated ROC curves, and the results showed no significant difference between the two prediction models. This conclusion may indicate that methods to evaluate coronary artery damage have little effect on the incidence of IVIG resistance.

The predictors of IVIG resistance in previous studies have included age, fever duration, delayed diagnosis, rash, oedema of extremities, days of illness at initial treatment, CRP, ESR, percentage of neutrophils, haemoglobin, neutrophil-to-lymphocyte ratio, platelet count, serum albumin, serum sodium, total bilirubin, serum ALT, serum AST, serum gamma-glutamyl transferase, serum interleukin-6, and abnormal echocardiogram [7,8,12,21,27–30]. In the present study, low haemoglobin, low serum albumin, high percentage of neutrophils, high CRP, high AST, the presence of coronary artery lesions, and complete Kawasaki disease were predictors of IVIG resistance. The findings for these variables were similar to the previous conclusions. However, a high platelet count and high serum sodium level might be high-risk factors for IVIG resistance, which was different from the findings of previous studies [7,30]. It is not known whether our results were applicable to other races or districts. On the other hand, patients with IVIG resistance show a severe inflammatory response [7], which may lead to reduced food intake, and reduced food intake and the relative increase in blood sodium and platelets caused by haemoconcentration may be one of the reasons.

To the best of our knowledge, serum alkaline phosphatase and complete Kawasaki disease have not been previously identified as predictors of IVIG resistance. We found that the coronary artery injury was significantly more common among IVIG-resistant children than in IVIG-responsive children; this was true of the training set and the two validation sets. This finding is contrary to Negar *et al.* [21]. The latter study was conducted in the United States. The contrary results were most likely due to racial differences between the study populations, but this must be confirmed in large multicentre studies.

The reason why ALP can predict IVIG resistance is still unknown. However, other previous studies on ALP may be helpful to explain this phenomenon. Lalle [31] reported that some type of alkaline phosphatase, like internal alkaline phosphatase, has been shown to be elevated in inflammatory disease necrotic enterocolitis (NEC). The previous study showed that human recombinant alkaline phosphatase (recAP) can reduce renal inflammation in septic acute kidney injury patients [32]. Because KD patients with IVIG unresponsiveness are thought to have severe inflammation and vasculitis [7], the higher ALP may be associated with a more severe inflammatory response. Peters et al. [32] reported the mechanism of this reason may be related to the dephosphorylation of adenosine triphosphate (ATP) and lipopolysaccharide (LPS). The probability for patients with high serum alkaline phosphatase to be at higher risk of IVIG resistance did not reach significance between IVIG-responsive and age-matched IVIG-resistant patients, probably because of the low number of observations in the IVIG-resistant group (*n* = 72, in a total of 1293 KD patients).

When the prediction model was compared with the model reported by Suzhou [12], the area under the ROC curvenin of this system was 0.79, similar to 0.75 of our system. Our model showed an AUC of 0.83 for prospective data, which was better than the previous system. In comparison with the model reported by Fuzhou [33], our model had better sensitivity (0.86 vs. 0.30), but poorer specificity (0.49 vs. 0.99) and a lower AUC area (0.66 vs. 0.74). Thus, in comparison with previous studies, our model shows similar applicability as the data obtained by Suzhou and Fuzhou. This conclusion also suggested that we only provided a new machine learning method to study IVIG resistance, and the prediction model of IVIG resistance needs to be further studied in the future.

### Limitations

The present study had several limitations. First, diagnoses were made by different doctors at the different centres; human errors in measurement are possible during cardiac colour Doppler ultrasound examination. Second, there are some missing data. Although the multiple imputation method was adopted, bias cannot be ruled out. Third, the data set for the LASSO regression analysis and the data set for external verification were from retrospective research, which is also prone to bias. Fourth, late data from subsequent prospectively enrolled patients were used to validate performance of the LASSO logistic model developed on the basis of the early data.

## Conclusions

The proposed nomogram—based on nine readily available clinical and laboratory parameters—appears to be a reliable predictor of IVIG resistance in patients hospitalized for Kawasaki disease in eastern China. Early identification of patients with risk of IVIG resistance will help in clinical decision making, such as use of additional corticosteroid therapy, during this critical period.

## Supplementary Material

Supplemental Material

## Data Availability

The data that support the findings of this study are openly available in figshare at DOI：10.6084/m9.figshare.15073134.
