# Supplementary material for: Nomogram to predict risk of resistance to intravenous immunoglobulin in children hospitalized with Kawasaki disease in Eastern China
Source: Ann Med. 2022 Jan 31;54(1):442–53. doi: 10.1080/07853890.2022.2031273 (PMC8812733; doi:10.1080/07853890.2022.2031273)
Supplement: Supplemental Material [file IANN_A_2031273_SM9849.zip › Supplemental files/supplement1.docx]

| Supplement 1 Missing values and imputation methods in the training set |
| --- |

| Variable | Type of model | Missing values | Percent of missing value |
| --- | --- | --- | --- |
| WBC count | Linear regression | 9 | 0.70% |
| Serum albumin | Linear regression | 12 | 0.93% |
| AST | Linear regression | 13 | 1.01% |
| % neutrophils | Linear regression | 14 | 1.08% |
| ALT | Linear regression | 15 | 1.16% |
| Platelet count | Linear regression | 26 | 2.01% |
| Tbil | Linear regression | 28 | 2.17% |
| Hemoglobin | Linear regression | 66 | 5.10% |
| CRP | Linear regression | 114 | 8.82% |
| Serum sodium | Linear regression | 329 | 25.44% |
| ALP | Linear regression | 346 | 26.80% |
| LDH | Linear regression | 588 | 45.50% |
| Serum calcium | None | 720 | 55.70% |
| Urine white blood cell count | None | 698 | 54.00% |

WBC: white blood cell; AST: serum aspartate aminotransferase; ATL: serum alanine aminotransferase; TBil: serum total bilirubin; CRP: C-reactive protein; ALP: serum alkaline phosphatase; LDH: serum lactate dehydrogenase.
