# Supplementary material for: Nomogram to predict risk of resistance to intravenous immunoglobulin in children hospitalized with Kawasaki disease in Eastern China
Source: Ann Med. 2022 Jan 31;54(1):442–53. doi: 10.1080/07853890.2022.2031273 (PMC8812733; doi:10.1080/07853890.2022.2031273)
Supplement: Supplemental Material [file IANN_A_2031273_SM9849.zip › Supplemental files/supplement2.docx]

| Supplement2 Variables are calculated in the training set using Random Forest |
| --- |

| Variable | meanImp | medianImp | minImp | maxImp | decision |
| --- | --- | --- | --- | --- | --- |
| sex | -0.03398 | -0.30931 | -1.18952 | 1.711232 | Rejected |
| age | 4.024649 | 4.11342 | 0.798005 | 6.86316 | Confirmed |
| CRP | 7.337426 | 7.439257 | 4.461535 | 10.17766 | Confirmed |
| WBC | 4.382191 | 4.311889 | 1.173786 | 6.715872 | Confirmed |
| HB | 7.466333 | 7.56547 | 4.208534 | 9.974634 | Confirmed |
| N | 7.227523 | 7.171567 | 4.485543 | 9.850382 | Confirmed |
| PLT | 1.281989 | 1.174822 | -0.15835 | 2.666724 | Rejected |
| ALB | 5.685708 | 5.834528 | 2.414652 | 8.177429 | Confirmed |
| AST | 3.374765 | 3.537758 | -0.15484 | 5.787283 | Confirmed |
| ALT | 2.890534 | 2.641303 | 0.34584 | 6.287745 | Confirmed |
| TBil | 2.941012 | 2.955405 | -0.37316 | 5.515616 | Confirmed |
| LDH | 0.182348 | 0.413071 | -1.08771 | 2.164493 | Rejected |
| Na | 4.40771 | 4.461825 | 0.536024 | 7.158864 | Confirmed |
| ALP | -0.05515 | -0.34608 | -1.54772 | 2.094879 | Rejected |
| CALs | 0.436359 | 0.077178 | -1.30469 | 2.488859 | Rejected |
| inKD | -0.03059 | -0.05668 | -1.74065 | 1.374132 | Rejected |

Imp: important; CRP: C-reactive protein; WBC: white blood cell; HB: hemoglobin; N: % neutrophils; PLT: Platelet count; ALB: Serum albumin ; AST: serum aspartate aminotransferase; ATL: serum alanine aminotransferase; TBil: serum total bilirubin; LDH: serum lactate dehydrogenase; Na: Serum sodium; ALP: serum alkaline phosphatase; CALs: coronary artery lesions; inKD: incomplete Kawasaki disease.
