# Supplementary figures and images for: Nomogram to predict risk of resistance to intravenous immunoglobulin in children hospitalized with Kawasaki disease in Eastern China
Source: Ann Med. 2022 Jan 31;54(1):442–53. doi: 10.1080/07853890.2022.2031273 (PMC8812733; doi:10.1080/07853890.2022.2031273)

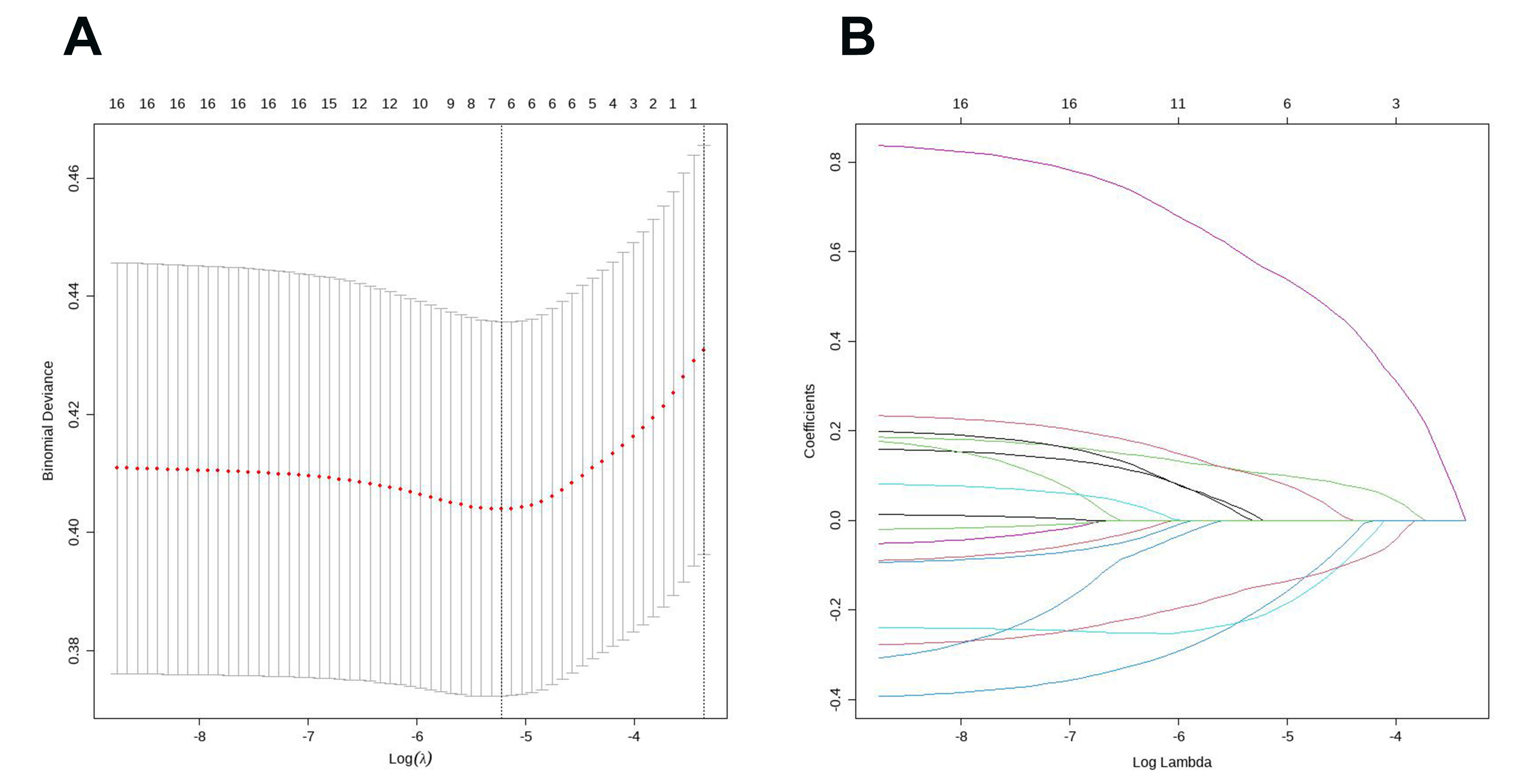

Supplement: Supplemental Material [file IANN_A_2031273_SM9849.zip › Supplemental files/Supplement 3.jpg]

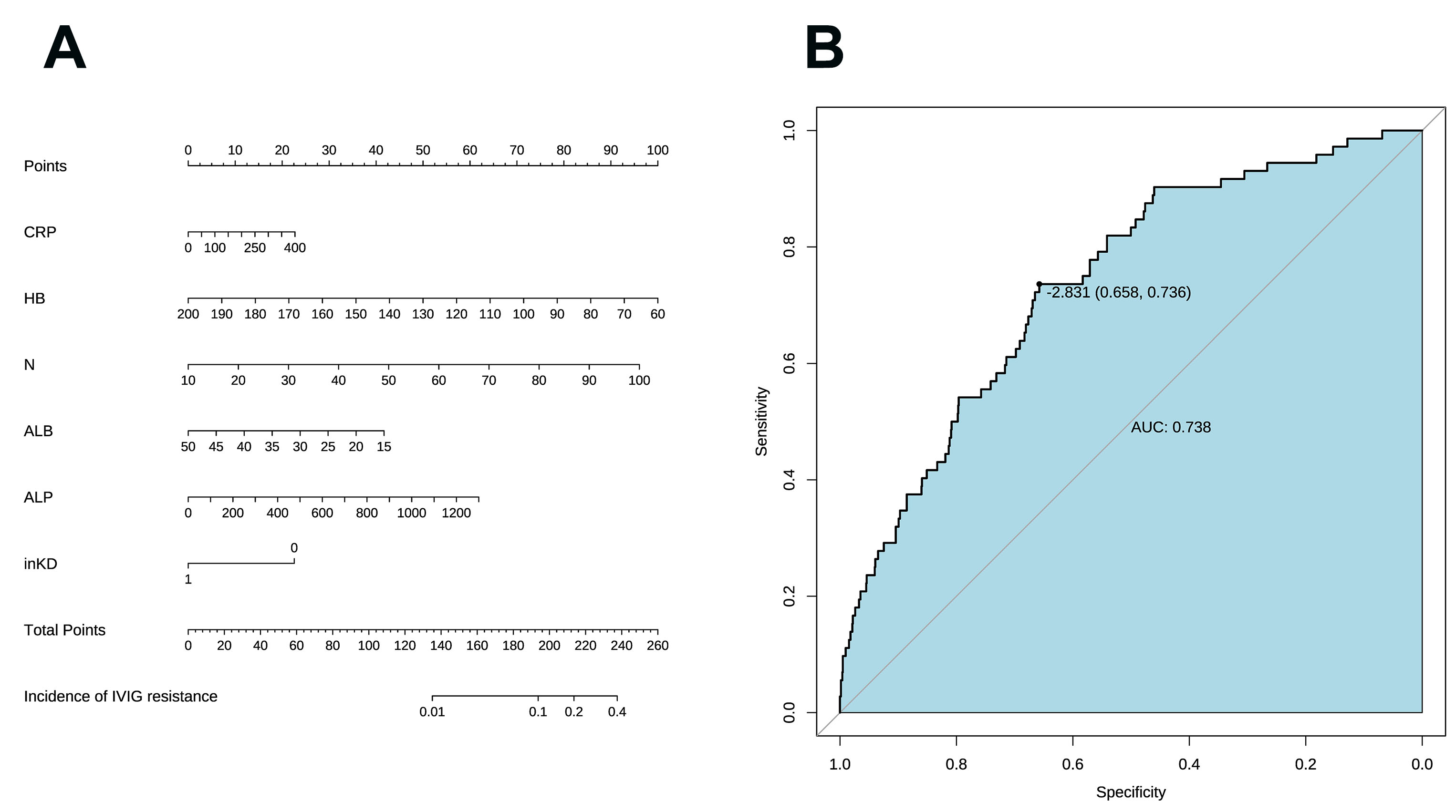

Supplement: Supplemental Material [file IANN_A_2031273_SM9849.zip › Supplemental files/supplement 4.jpg]
